# Plummer Vinson Syndrome: A Rare Syndrome in Male with Review of the Literature

**DOI:** 10.1155/2017/6205925

**Published:** 2017-08-28

**Authors:** Priyadarshini Karthikeyan, Nalini Aswath, Ramesh Kumaresan

**Affiliations:** ^1^Department of Oral Medicine and Radiology, AIMST University, Faculty of Dentistry, Jalan Semeling-Bedong, 08100 Bedong, Kedah, Malaysia; ^2^Department of Oral Medicine and Radiology, Sree Balaji Dental College and Hospitals, Chennai, Tamil Nadu, India; ^3^Academic Unit of Craniofacial Clinical Care, AIMST University, Faculty of Dentistry, Jalan Semeling-Bedong, 08100 Bedong, Kedah, Malaysia

## Abstract

**Introduction:**

Plummer Vinson syndrome also known as Paterson Brown-Kelly syndrome is a syndrome associated with the triad of symptoms comprising microcytic hypochromic anemia, oesophageal strictures, and dysphagia. PVS is commonly found in women of middle age especially in the fourth and fifth decade of life and is rarely reported in males.

**Case Report:**

The authors report a case of 43-year-old male patient who presented with the classic symptoms of Plummer Vinson syndrome.

**Conclusion:**

Dentists have to be familiar with symptoms of PVS and a thorough clinical examination of the patient is necessary for early diagnosis and treatment. As PVS is a precancerous condition with high malignant potential, early diagnosis is of utmost importance for better prognosis.

**Clinical Significance:**

Mutual interaction of systemic and oral health has largely been underestimated by many patients in the developing countries and hence this report includes a note on importance of adequate medical history taking and its relevance to the dental health and treatment.

## 1. Introduction

A wide range of systemic disorders has their manifestations in the orofacial region. Though, most of these manifestations are nonspecific they should alert the dental surgeon to the possibility of an underlying systemic disorder. These manifestations should be properly recognized before the patient receives appropriate diagnosis and referral for treatment. The importance of understanding these manifestations also lies in the fact that these orofacial signs and symptoms may be the first or the only clinical presentation that may alert the dental surgeon regarding an underlying systemic disorder [1].

Plummer Vinson syndrome (PVS) is one such syndrome characterized by a triad of chronic iron deficiency anemia, dysphagia, and esophageal webs. PVS mainly affects white women in the 4th–7th decade [2]. It has been rarely described in males. The diagnosis of PVS relies on thorough history taking, general clinical examination, hematological investigation, and radiological examination. However, in developing country, many patients underestimate the relevance of medical history to dental health and treatment. In this case report we present a male patient, who presented with classical symptoms of PVS; however, the patient presumed that it was not necessary for the dentist to know about his complete medical history and hence did not reveal these symptoms during initial examination.

## 2. Case Report

**Figure 1:**
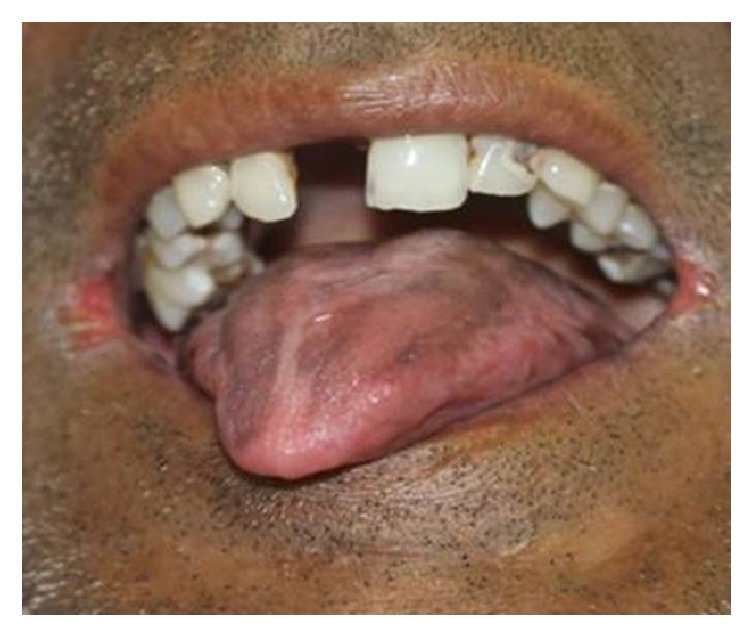
Male patient with glossitis and angular cheilitis.

A 43-year-old male patient reported to the dental outpatient department with the complaint of mobility of the lower front teeth since two years. Patient gave a history of trauma two years back due to which the lower front teeth became mobile. His medical and personal histories were noncontributing. On general examination, patient's palpebral conjunctiva was pale, signifying anemia and the finger nails were spoon shaped (koilonychia). Intraoral examination revealed difficulty in mouth opening and presence of ulcers at the corners of the mouth with pigmentations on the tongue in addition to mobile lower anterior teeth (Figure 1). When inquired about the ulcers, the patient gave history of recurrent ulcers at angle of mouth for past one year.

Peripheral smear study revealed microcytic, hypochromic anemia and laboratory investigations were significant for a profound iron deficiency anemia (Table 1). Since no definitive cause for iron deficiency was evident, a detailed anemia history was taken and the patient complained of easy fatigability and weight loss since five months and also had difficulty and burning sensation while swallowing liquid and solid foods. When further inquired about not revealing these symptoms during the initial history taking phase, the patient responded that he was unaware of their relevance to the dental treatment and presumed that it was not necessary for the dentist to know about his complete medical history.

**Figure 2:**
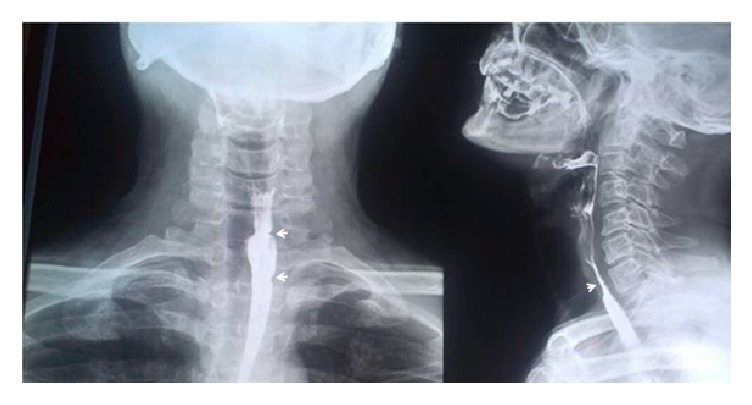
Barium esophagram showing the presence of Cervical-Esophageal Web.

Subsequently, a radiograph examination which consisted of barium swallow test was done which revealed the constriction of esophagus. A smooth narrowing was noted in the lateral aspect of cervical esophagus. Partial web is seen suggestive of Cervical-Esophageal Web (Figure 2). The clinical, hematological, and radiographic findings fulfil the triad of PVS. The patient was treated with oral iron supplements of 120 mg/day with dietary advice. The patient showed a significant improvement in his symptoms by the end of eight weeks of treatment.

## 3. Discussion

### 3.1. History

In 1912, Henry Stanley Plummer reported a series of patients with dysphagia who presented with diffuse dilation of the esophagus and spasm of upper esophagus without anatomical stenosis, which was described as hysterical or neurosis of unknown origin [3]. Subsequently, Porter Paisley Vinson in 1919 reported a relation between the dysphagia and angulation of the esophagus and also stated that this type of dysphagia presented with three characteristic manifestations: anemia, dysphagia, and atrophic glossitis and attributed the first description of this entity to the earlier report of Plummer [1]. Since then, the syndrome associated with these symptoms has been called Plummer Vinson syndrome. PVS is also termed as Paterson-Kelly syndrome, named after Donald Ross Paterson and Adam Brown-Kelly, who first described the characteristic clinical features of the syndrome and published their findings independently in 1919 [4]. This syndrome is also known as Sideropenic dysphagia which implies the burning sensation or throat pain while swallowing. The radiological features of this syndrome was described by Jan Waldenstrom and Sven Roland Kjellberg in 1939, who gave specific and objective signs as a basis for the roentgenological diagnosis of this syndrome. Hence, the syndrome is also termed as Waldenstrom-Kjellberg syndrome [5].

### 3.2. Epidemiology

The data regarding the incidence and prevalence of the syndrome are not evidently available and few data are not reliable as hematological parameters are not included [6]. Only case reports and few case series have been published in literature. In the early 20th century PVS seemed to be common in Caucasians of Northern countries, particularly among middle-aged women, with the mean age at presentation being 47 years (range 28–80 years) [4]. However, some pediatric and adolescent cases were also reported [6]. PVS has a remarkably high female to male ratio of 4 : 1. Recently, few reports of PVS in male patient are being published leading to notion that PVS might be common in both males and females [3]. Our case report also documents a middle-aged male patient who presented with clinical features of PVS. However, it is considered that PVS has become increasingly rare nowadays with improvement of nutritional status in the developing countries and also due to the availability of iron supplements; nonetheless it should be suspected in cases of iron deficiency and dysphagia due to its malignant potential [7].

### 3.3. Etiopathogenesis

The exact etiopathogenesis of the PVS remains controversial. But iron deficiency anemia, nutritional deficiencies, genetic predisposition, and autoimmune etiologies may be the contributing factors. Iron deficiency anemia is most widely accepted etiology as the symptoms are improved with iron supplementation as also noted in our patient [1]. Iron deficiency causes rapid depletion of iron-dependent oxidative enzymes which results in myasthenic changes in muscles of alimentary tract, causing mucosal degeneration, muscle atrophy, and esophageal web formation, and also leads to neoplastic changes of the pharynx and upper esophagus. An autoimmune mechanism is positively associated as PVS, as it has been reported in association with rheumatoid arthritis, celiac disease, pernicious anemia, and thyroiditis [2].

### 3.4. Clinical Features and Oral Manifestations

The main clinical features of PVS include the triad of iron deficiency anemia, cervical dysphagia and esophageal webs. The dysphagia is usually painless and initially limited to solids and sometimes associated with weight loss. Over time, dysphagia can progress to involve liquids as well. The progressive dysphagia is often tolerated by the patients for considerable period of time without seeking medical attention, leading to a late presentation [8]. This might be the reason why our patient did not seek medical attention for a long time.

Esophageal web is a thin 2-3 mm membrane, covered with pink mucosa, consisting of mucosa and submucosa without the muscle layer, usually occurring in the proximal 4-5 cm of esophagus. These esophageal webs are usually eccentric, semilunar, or annular and present with dysphagia if the diameter of the lumen through the web is less than 12 mm [3].

Patients also usually complain of anemic symptoms such as weakness, pallor, fatigue, and tachycardia. Other features may include esophagitis, cardiospasm, achlorhydria, nail deformation that includes koilonychias or clubbing, enlargement of spleen and thyroid, splenic tumors, dermatitis, seborrheica, hyperkeratosis, conjunctivitis, keratitis, blepharitis, and visual disturbances [1].

Oral manifestations in patients with Plummer Vinson syndrome includes all the features of iron deficiency anemia like stomatitis, glossitis, angular cheilitis, erythematous mucositis, recurrent aphthous stomatitis, pale oral mucosa, oral candidiasis, dry mouth in 49.3% of patients, burning mouth in 76–100% of patients, lingual varicosity in 56%, oral lichen planus in 33.3%, recurrent aphthous ulceration in 25.33%, and early loss of teeth. Due to nutritional deficiency in these patients, the filiform papillae disappear first followed by fungiform papillae; regeneration of papillae occurs in reverse order but vallate and foliate papillae on the posterior third are spared. It also leads to taste dysfunction in 12% of patients [1, 2, 4, 9].

In the present case, the patient suffered from dysphagia, ulceration, and burning sensation in his mouth, glossitis, angular cheilitis, and koilonychias which included most of the common oral manifestation.

### 3.5. Laboratory Tests

Hematological investigation typically reveals iron deficiency anemia with decreased values of hemoglobin, hematocrit, MCV, and serum ferritin and increased total iron binding capacity. Peripheral smear study reveals microcytic hypochromic anemia. Few authors suggest thyroid profile to rule out hypothyroidism as thyroid hormones are involved in hemoglobin synthesis and hence may lead to anemia [10]. Hematological investigations in our patient were significant for a profound iron deficiency anemia.

### 3.6. Radiographic Examination

Esophageal webs and strictures can be detected by radiographic methods or endoscopy. However, radiographic method is more suitable as endoscopy can sometimes miss the point of benign stricture and does not detect most of the motility disorders [11]. Barium swallow test is the most sensitive test to diagnose esophageal webs. Barium sulfate is an inert material that produces good contrast; however, for better visualization a thick paste should be used with rapid exposure after swallowing [10]. In our patient barium swallow test revealed a smooth narrowing in the lateral aspect of cervical esophagus suggestive of Cervical-Esophageal Web.

### 3.7. Diagnosis

The diagnosis of PVS is based on the evidence of iron deficiency anemia and one or more esophageal web in a patient with postcricoid dysphagia. Hence, the diagnosis relies on thorough history taking, general clinical examination, hematological investigation (anemia profile), and radiological examination (plain lateral X-ray on the neck and barium swallow test). Supplementary investigation like direct endoscopic examination, videofluoroscopy, and biopsy taking for histopathological examination might be required in few patients [4]. Our patient presented with the classic triad and also hematological and radiological investigation are suggestive of PVS.

### 3.8. Differential Diagnosis

Differential diagnosis of PVS includes all the likely causes of dysphagia like malignant tumors, spastic motility disorders, benign strictures, scleroderma, diverticula, achalasia, gastroesophageal reflux disease, esophageal burns, skeletal muscle disorders, and neuromuscular disorders [5, 7].

### 3.9. Malignant Potential

PVS has been identified as a risk factor for developing squamous cell carcinoma of the upper aerodigestive tract in 3%–15% of patients mostly in women between 15 and 50 years of age and almost occurs in the postcricoid region. The syndrome is considered to be a precancerous condition because squamous cell carcinoma of hypopharynx, upper esophagus, or oral cavity takes place in 10% of these patients. The mechanism explained is that the anemia causes epithelial atrophy and decreases the repair capacity of the mucosa which allows the carcinogens and cocarcinogens to act aggressively, predisposing the entire oral cavity and esophageal area to malignancy [7, 10]. A rare association of this syndrome with base of tongue cancers has been reported in literature [6]. Hence, these patients should be followed up by upper gastrointestinal endoscopies to assess any neoplastic changes. Cytogenetic assessment has been suggested as a good predictor of the possibility of postcricoid carcinoma.

### 3.10. Management

Initial step in the management of PVS is to detect the cause of iron deficiency. In females it is mostly due to increased menstrual flow and in males an underlying malignancy should be ruled out. Management consists of correcting underlying iron deficiency anemia, iron supplements, and mechanical dilatation of the webs. Many studies have shown that iron supplementation alone can improve the symptoms and no mechanical dilatation was required [2]. This holds true in our case in whom the symptoms improved on iron therapy.

Mechanical dilatation of webs can be carried out by endoscopic bougies or pneumatic balloons in single or multiple sessions. Surgery is reserved only for recalcitrant webs. Other rare means of disruption of web include ND:YAG laser therapy or needle-knife electroincision [4, 7, 10].

The patient with PVS should be kept on a nutritious diet to maintain the integrity and maturative potential of the oral epithelium due to its malignant potential [7].

### 3.11. Prognosis

General prognosis of this condition is good as anemia and dysphagia can be effectively treated with iron supplementation either alone or along with web dilatation. However, prognosis worsens drastically if associated with malignant disorder. A surveillance upper gastrointestinal endoscopy is recommended every year [4].

### 3.12. Importance of Adequate Medical History Taking

A patient's medical history is a vital part of his or her dental history and increases the dentist's awareness of diseases and medication which might interfere with the patient's diagnosis and dental treatment. The mutual interaction of systemic and oral health has largely been underestimated by many patients in the developing countries as it is clearly evident in our case. Adequate time has to be allotted to understand why a patient might not want to reveal his complete medical history; besides earnest effort has to be taken to inform them about the importance of their medical history and its relevance to their dental health and treatment. However, the lone way to satisfactorily get the patient to reveal their full medical history is by practicing the art of interviewing and clinical physical examination.

## 4. Conclusion

Mouth is the diagnostic mirror of various systemic diseases. Many systemic diseases manifest in the oral cavity and mouth can show early signs or the only signs of a disease process at a site elsewhere. PVS is one such systemic condition with predominant oral symptoms. Hence, dentists have to be familiar with symptoms of PVS and a thorough clinical examination of the patient is necessary for early diagnosis and treatment. As PVS is a precancerous condition with high malignant potential, early diagnosis is of utmost importance for better prognosis. Also, dentists should practice the art of interviewing the patient towards revealing their full medical history and perform an effective and complete clinical examination.

## Figures and Tables

**Table 1:** Laboratory results.

	Patient laboratory result	Normal laboratory range
Red blood cell count	2.1 cells/mcL	4.0–6.0 cells/mcL
Hemoglobin	6.5 g/dL	13–17 g/dL
Mean corpuscular hemoglobin	15.1 pg/dL	27–31 pg/dL
Mean corpuscular hemoglobin concentration	28.9 g/dL	32–36 g/dL
Mean cell volume	52.0 fl	80.0–99.0 fl
Serum iron	20 *μ*g/dL	50–150 *μ*g/dL
Total iron-binding capacity	548 *μ*g/dL	250–450 *μ*g/dL
Serum ferritin	3.26 ng/mL	23–336 ng/mL
White blood cell count	5.3 k/mm^3^	4.5–10.0 k/mm^3^
Vitamin B12 level	374.8 pg/mL	200–900 pg/mL
